# Supplementary material for: Anti-SARS-CoV-2 antibody kinetics up to 6 months of follow-up: Result from a nation-wide population-based, age stratified sero-epidemiological prospective cohort study in India
Source: PLoS One. 2023 Dec 11;18(12):e0287807. doi: 10.1371/journal.pone.0287807 (PMC10712846; doi:10.1371/journal.pone.0287807)
Supplement: S1 File — (DOCX) [file pone.0287807.s001.docx]

**S1 to S6 tables on the supporting information of study result**

**S1 Table. The period of the data collection according to the study site and area**

| **Study Site** | **Area** | **Round One** | **Round Two** | **Round Three** |
| --- | --- | --- | --- | --- |
| **AIIMS, New Delhi** | **Rural** | March 3, 2021 – June 10, 2021 | May 27, 2021 – Oct 18, 2021 | August 26, 2021 – Dec 31, 2021 |
|  | **Urban** | March 15, 2021 – July 31, 2021 | June 18, 2021 – Oct 26, 2021 | Sept 28, 2021 – Dec 11, 2021 |
| **AIIMS, Bhubaneswar** | **Rural** | March 22, 2021 – May 7, 2021 | July 9, 2021 – August 11, 2021 | Oct 11, 2021 – Dec 19, 2021 |
|  | **Urban** | June 7, 2021 – June 30, 2021 | Sept 6, 2021 – Oct 11, 2021 | Oct 12, 2021 – Jan 14, 2022 |
| **AIIMS, Gorakhpur** | **Rural** | April 22, 2021 - June 29, 2021 | July 24, 2021 – Oct 4, 2021 | Oct 12, 2021 – Dec 19, 2021 |
|  | **Urban** | July 1, 2021 – August 18, 2021 | Oct 5, 2021 – Nov 27, 2021 | Dec 18, 2021 – Jan 10, 2022 |
| **Agartala Medical College** | **Rural** | 26 March 2021 – June 1, 2021 | July 12, 2021 – Dec 10, 2021 | Sept 20, 2021 – Dec 31, 2021 |
|  | **Tribal** | 17 June 2021 – August 7, 2021 | Sept 14, 2021 – Nov 29, 2021 | Dec 15, 2021 – Dec 31, 2021 |
| **JIPMER, Pondicherry** | **Rural** | June 9, 2021 – July 30, 2021 | Sept 1, 2021 – Nov 23, 2021 | Oct 12, 2021 – Dec 27, 2021 |
|  | **Urban** | June 10, 2021 – July 23, 2021 | Sept 2, 2021 – Nov 27, 2021 | Oct 12, 2021 – Dec 28, 2021 |

**S2 Table. Distribution of participants by to the study site, round, and area**

| **Study site** | **Area** | **Round One**  **n (%)** | | **Round Two**  **n (%)** | | **Round Three**  **n (%)** | |
| --- | --- | --- | --- | --- | --- | --- | --- |
|  |  | **Total** | **Area wise^*^** | **Total** | **Area wise** | **Total** | **Area wise** |
| **Delhi** | **Urban** | 2060  (100%) | 1001  (47.2%) | 1569  (76.2%) | 741/1001 (74.0%) | 1395  (67.7%) | 780/1001 (77.9%) |
|  | **Rural** |  | 1059  (52.8%) |  | 828/1059 (78.2%) |  | 708/1395 (66.8%) |
| **Bhubaneswar** | **Urban** | 2000  (100%) | 1000  (50.0%) | 1704  (85.2%) | 820/1000 (82.0%) | 1473  (73.6%) | 777/1000 (77.7%) |
|  | **Rural** |  | 1000  (50.0%) |  | 884/1000 (88.4%) |  | 700/1000 (70.0%) |
| **Gorakhpur** | **Urban** | 2010  (100%) | 1002  (49.8%) | 1151  (57.3%) | 553/1002 (55.2%) | 937  (46.6%) | 509/940  (50.8%) |
|  | **Rural** |  | 1008  (50.2%) |  | 598/1008 (59.3%) |  | 431/1008  (42.7%) |
| **Agartala** | **Tribal** | 2000  (100%) | 1339  (66.9%) | 856  (42.8%) | 489/1339  (36.5%) | 1095  (54.7%) | 672/1339 (31.7%) |
|  | **Rural** |  | 661  (33.1%) |  | 368/661  (55.6%) |  | 423/639  (63.9%) |
| **Pondicherry** | **Urban** | 2040  (100%) | 1020  (50.0%) | 1222  (59.9%) | 601/1020 (49.2%) | 664  (33.1%) | 322/1020  (31.6%) |
|  | **Rural** |  | 1020  (50.0%) |  | 621/1020 (60.1%) |  | 352/1020  (34.5%) |
| **Total** | **Urban** | 10110  (100%) | 4023  (39.7%) | 6503  (64.3%) | 2715/4023  (67.5%) | 5564  (55.0%) | 2164/4023  (53.7%) |
|  | **Rural** |  | 4748  (46.9%) |  | 3229/4748  (69.5%) |  | 2728/4748  (57.4%) |
|  | **Tribal** |  | 1339  (13.2%) |  | 489/1339  (36.5%) |  | 672/1339  (50.2%) |

*Column % is given whereas for all other figures, the denominator is the number of round one

**S3 Table. Distribution of total participants in urban and rural area by survey round, sex, and age group**

| **Variable** | | **Rural n (%)** | | | **Urban n (%)** | | | **Tribal n (%)** | | |
| --- | --- | --- | --- | --- | --- | --- | --- | --- | --- | --- |
|  |  | **Round One**  **(n=4748)** | **Round Two**  **(n=3299)** | **Round Three**  **(n=2728)** | **Round One**  **(n=4023)** | **Round Two**  **(n=2715)** | **Round Three**  **(n=2164)** | **Round One**  **(n=1339)** | **Round Two**  **(n=489)** | **Round Three**  **(n=672)** |
| **Sex** | **Male** | 2060 (43.4) | 1349 (40.9) | 1057 (38.7) | 1906 (47.4) | 1240 (45.7) | 956  (44.2) | 554 (41.4) | 178 (36.4) | 262 (38.9) |
|  | **Female** | 2688 (56.6) | 1950 (59.1) | 1671 (61.3) | 2117 ((52.6) | 1475 (54.3) | 1208 (55.8) | 785 (58.6) | 311 (63.6) | 410 (61.1) |
| **Age Group** | **1-4 years** | 35  (0.7) | 25  (0.7) | 20  (0.7) | 10  (0.3) | 5  (0.2) | 4  (0.2) | 0 | 0 | 0 |
|  | **5-9 years** | 179 (3.4) | 120  (3.6) | 101  (3.7) | 130  (3.2) | 83  (3.1) | 64  (2.9) | 13  (0.9) | 4  (0.8) | 6  (0.9) |
|  | **10-14 years** | 386 (8.1) | 276  (8.4) | 225  (8.3) | 283  (7.0) | 195  (7.2) | 148  (6.8) | 50  (3.7) | 7  (1.4) | 21  (3.1) |
|  | **15-19 years** | 430 (9.1) | 291  (8.8) | 231  (8.5) | 285  (7.1) | 180  (6.6) | 114  (5.3) | 80  (5.9) | 13  (2.6) | 30  (4.5) |
|  | **20-29 years** | 747 (15.7) | 473 (14.3) | 389 (14.3) | 581  (14.4) | 348 (12.8) | 247  (11.4) | 242  (18.1) | 74  (15.1) | 92  (13.7) |
|  | **30-39 years** | 795 (16.7) | 549 (16.7) | 450 (16.5) | 638  (15.8) | 412 (15.2) | 353  (16.3) | 290  (21.6) | 108 (22.1) | 173 (25.7) |
|  | **40-49 years** | 784 (16.5) | 550 (16.7) | 476 (17.5) | 748 (18.6) | 502 (18.5) | 414  (19.1) | 261  (19.5) | 99  (20.3) | 139 (20.7) |
|  | **50-59 years** | 616 (12.9) | 428 (12.9) | 357 (13.1) | 621  (15.4) | 442 (16.3) | 353  (16.3) | 188  (14.0) | 92  (18.8) | 96  (14.3) |
|  | **60-69 years** | 475 (10.0) | 359 (10.8) | 292 (10.7) | 492  (12.2) | 371 (13.6) | 323  (14.9) | 129  (9.6) | 57  (11.6) | 75  (11.9) |
|  | **70+ years** | 301  (6.3) | 228  (6.9) | 187  (6.8) | 235  (5.8) | 177  (6.5) | 144  (6.7) | 86  (6.4) | 35  (7.2) | 40  (5.9) |

**S4 Table. Distribution of reasons for loss to follow up participants by round area**

| **Reasons for loss to follow up** | **Urban** | | **Rural** | | **Tribal** | |
| --- | --- | --- | --- | --- | --- | --- |
|  | **Round Two**  **n= 1308 (%)** | **Round Three**  **n= 1859 (%)** | **Round Two**  **n= 1449 (%)** | **Round Three**  **n= 2020 (%)** | **Round Two**  **n= 850 (%)** | **Round Three**  **n= 667 (%)** |
| **Refused** | 487 (37.2) | 873 (46.9) | 685 (47.3) | 1084 (53.7) | 841 (98.9) | 665 (99.7) |
| **Deceased** | 8 (0.6) | 13 (0.7) | 9 (0.6) | 14 (0.7) | 2 (0.2) | 2 (0.2) |
| **Sick at the time of visit** | 158 (12.1) | 335 (18.0) | 286 (19.7) | 379 (18.7)) | 7 (0.8) | 0 |
| **Could not be found at home after three visit** | 655 (50.1) | 638 (34.3) | 469 (32.3) | 543 (26.8) | 0 | 0 |

**S5 Table. Distribution of SARS-CoV-2 seropositive of participants by site, round and area**

| **Sites** | **Area** | **Round One** | | **Round Two** | | **Round Three** | |
| --- | --- | --- | --- | --- | --- | --- | --- |
| **Delhi** | **Urban** | 1373 (66.6) | 749 (74.8) | 1485 (94.6) | 721 (97.3) | 1336 (95.8) | 679 (98.4) |
|  | **Rural** |  | 624 (58.9) |  | 764 (92.3) |  | 657 (93.2) |
| **Bhubaneswar** | **Urban** | 1283 (64.1) | 732 (73.2) | 1428 (83.0) | 669 (81.6) | 1284 (87.2) | 566 (80.9) |
|  | **Rural** |  | 551 (55.1) |  | 759 (85.8) |  | 718 (92.7) |
| **Gorakhpur** | **Urban** | 1845 (91.7) | 948 (94.6) | 1093 (94.9) | 537 (97.1) | 903 (96.4) | 420 (97.7) |
|  | **Rural** |  | 897 (88.9) |  | 556 (92.9) |  | 483 (95.3) |
| **Agartala** | **Tribal** | 1254 (62.7) | 893 (66.7) | 777  (90.7) | 442 (90.4) | 1020 (93.2) | 615 (91.6) |
|  | **Rural** |  | 361 (54.6) |  | 335 (91.0) |  | 405 (95.5) |
| **Pondicherry** | **Urban** | 1719 (84.2) | 849 (83.2) | 1114 (91.2) | 545 (90.6) | 627 (94.4) | 323 (93.6) |
|  | **Rural** |  | 870 (85.3) |  | 569 (91.6) |  | 304 (95.3) |
| **Total** | **Urban** | 7474 (73.9) | 3278 (81.5) | 5897 (90.7) | 2472 (91.1) | 5170 (92.9) | 1988 (91.8) |
|  | **Rural** |  | 3303 (69.6) |  | 2983 (90.4) |  | 2567 (94.1) |
|  | **Tribal** |  | 893 (66.7) |  | 442 (90.4) |  | 615 (91.6) |

**S6 Table. Distribution of SARS-CoV-2 seropositive of participants by round, symptom status and site**

| Round | Symptom status | Agartala | | Bhubaneshwar | | Delhi | | Gorakhpur | | Puducherry | | Total | |
| --- | --- | --- | --- | --- | --- | --- | --- | --- | --- | --- | --- | --- | --- |
|  |  | Sero-positive | n (%) | Sero-positive | n (%) | Sero-positive | n (%) | Sero-positive | n (%) | Sero-positive | n (%) | Sero-positive | n (%) |
| Round one | **Symptoms Positive** | 1254 | 339 (27.0) | 1283 | 384 (29.9) | 1373 | 443 (32.3) | 1845 | 569 (30.8) | 1719 | 247 (14.4) | 7474 | 1982 (26.5) |
|  | **Symptoms Negative** |  | 915 (72.9) |  | 899 (70.0) |  | 930 (67.7) |  | 1276 (69.2) |  | 1472 (85.6) |  | 5492 (73.5) |
| Round Two | **Symptoms Positive** | 776 | 290 (37.4) | 1429 | 248 (17.4) | 1485 | 255 (17.2) | 1093 | 612 (55.9) | 1114 | 72 (6.5) | 5897 | 1477 (25.1) |
|  | **Symptoms Negative** |  | 486 (62.6) |  | 1181 (82.6) |  | 1230 (82.8) |  | 481 (44.1) |  | 1042 (93.5) |  | 4420 (74.9) |
| Round Three | **Symptoms Positive** | 1020 | 139 (13.6) | 1284 | 136 (10.6) | 1336 | 249 (18.6) | 903 | 459 (50.8) | 627 | 54 (8.6) | 5170 | 1037 (20.1) |
|  | **Symptoms Negative** |  | 881 (86.4) |  | 1148 (89.4) |  | 1087 (81.4) |  | 444 (49.2) |  | 573 (91.4) |  | 4133 (79.9) |
