# Supplementary material for: Anti-SARS-CoV-2 antibody kinetics up to 6 months of follow-up: Result from a nation-wide population-based, age stratified sero-epidemiological prospective cohort study in India
Source: PLoS One. 2023 Dec 11;18(12):e0287807. doi: 10.1371/journal.pone.0287807 (PMC10712846; doi:10.1371/journal.pone.0287807)
Supplement: Attachment — Submitted filename: Response to Reviewers 15 Nov.pdf [file pone.0287807.s003.pdf]

Point to point reply to editorial comments

Manuscript Title: Anti-SARS-CoV-2 Antibody Kinetics up to 6 months of follow-up: Result from a Nation-wide Population-based, Age Stratified Sero-Epidemiological Prospective Cohort Study in India

Manuscript ID: PONE-D-23-16775

Comments for the journal requirement:

**Comments**	**Revision/Reply**
We note that you have indicated that data from this study are available upon request. Please note PLOS only allows data to be available upon request if there are legal or ethical restrictions on sharing data publicly.<br><br>In this instance it seems there may be acceptable restrictions in place, however we ask that you please kindly clarify in detail the reasons for data restriction (e.g., data contain potentially sensitive information, etc.) and who has imposed them (e.g., an ethics committee). Please also provide a non-author point of contact (e.g., data access committee, ethics committee, or other institutional body) where data requests may be made. Note that it is not acceptable for the authors to be the sole named individuals responsible for ensuring data access.	The data set has identifiable information of family and patients along with lot of medical history which are potentially sensitive.<br><br>**The restriction has been imposed by:**<br>Institute Ethics Committee<br><br>Therefore, the data may be requested with the corresponding author Dr Puneet Misra : doctormisra@gmail.com or with the Dean (Research) of the investigating institute to the following address:<br><br>**Non-author point of contact:**<br>Dr. Jeewan Singh Titiyal<br>Dean, Research<br>Research Section<br>All India Institute of Medical Sciences,<br>Ansari Nagar East<br>New Delhi-110029<br>Mail:

	researchsectionaiims@gmail.com
