# Supplementary material for: Anti-SARS-CoV-2 antibody kinetics up to 6 months of follow-up: Result from a nation-wide population-based, age stratified sero-epidemiological prospective cohort study in India
Source: PLoS One. 2023 Dec 11;18(12):e0287807. doi: 10.1371/journal.pone.0287807 (PMC10712846; doi:10.1371/journal.pone.0287807)
Supplement: Attachment — Submitted filename: Reveiwer comments.docx [file pone.0287807.s002.docx]

Anti SARS-CoV-2 Antibody Kinetics up to 6 months of follow-up: Result from a Nation-wide Population-based, Age Stratified Sero-Epidemiological Prospective Cohort Study in India

The aim of the study was: Therefore, we aimed to assess the change in antibody prevalence and its kinetics in a closed cohort over six months across the country in different sub-population in India.

The authors have concluded that: There is a further need for study for longer follow-up and finding actual protection from subsequent infection.

A clarification is required that did the authors assess any protection from subsequent infection in their study? If it is not assessed then this conclusion needs to be deleted.
